# Fungal Diversity in a Dark Oligotrophic Volcanic Ecosystem (DOVE) on Mount Erebus, Antarctica

**DOI:** 10.3390/biology2020798

**Published:** 2013-05-30

**Authors:** Laurie Connell, Hubert Staudigel

**Affiliations:** 1 School of Marine Sciences, University of Maine, Orono, ME 04496, USA; 2 Institute of Geophysics and Planetary Physics, Scripps Institution of Oceanography, La Jolla, CA 92093, USA; E-Mail: hstaudig@ucsd.edu

**Keywords:** Antarctica, fungal community, volcano, Mt. Erebus

## Abstract

Fumarolic Ice caves on Antarctica’s Mt. Erebus contain a dark oligotrophic volcanic ecosystem (DOVE) and represent a deep biosphere habitat that can provide insight into microbial communities that utilize energy sources other than photosynthesis. The community assembly and role of fungi in these environments remains largely unknown. However, these habitats could be relatively easily contaminated during human visits. Sixty-one species of fungi were identified from soil clone libraries originating from Warren Cave, a DOVE on Mt. Erebus. The species diversity was greater than has been found in the nearby McMurdo Dry Valleys oligotrophic soil. A relatively large proportion of the clones represented *Malassezia* species (37% of Basidomycota identified). These fungi are associated with skin surfaces of animals and require high lipid content for growth, indicating that contamination may have occurred through the few and episodic human visits in this particular cave. These findings highlight the importance of fungi to DOVE environments as well as their potential use for identifying contamination by humans. The latter offers compelling evidence suggesting more strict management of these valuable research areas.

## 1. Introduction

The subsurface biosphere has been among the most exciting, and rapidly evolving research ecosystem types in biogeosciences of the past 20 years [1]. Trace fossils of microbial dissolution in seafloor volcanic rocks suggest the presence of a Dark Oligotrophic Volcanic Ecosystem (DOVE) at least for the upper 500 m and extending back to first appearance of life on Planet Earth [2]. DOVEs take a special role in the study of the subsurface biosphere. They are volumetrically very significant and they contain abundant energy donors from the earth’s interior, in the form of minerals and glasses that are highly reactive in low temperature hydrous environments. DOVEs commonly have active hydrothermal systems that readily circulate surface water and atmospheric gases through the interior of these volcanoes. The combination of surface-derived fluids and volcanic rocks from the interior of the earth providing abundant and effective combinations of electron acceptors and donors that can facilitate chemolithoautotrophic conditions for microbial communities to thrive without photosynthesis. Conditions of (near-) atmospheric oxygenation exist in very large fractions of DOVEs where water or air circulates relatively freely and recharges hydrothermal systems in active systems. These factors combine to make DOVEs a significant component of the subsurface biosphere and open up the possibility that DOVEs might provide biomass to the earth’s surface offering a “rock bottom” for the food web.

The southernmost active volcano in the world, Mt. Erebus (3,795 m), has been the focus of research for decades [3]. Recently, ice caves and fumarolic ice towers near the summit have been recognized as a unique environment devoid of light and/or having a moist warm environment [4]. These sub-glacial fumaroles issue gases that are dominated by air with 80–100% humidity and up to 2% CO_2_ [3,4]. CO_2_ is one of the few sources of carbon available to these microbial communities, although small amounts of organic carbon may enter the cave through melt water from the surface during the summer months that can contain algae or wind delivered carbon sources.

Some of the Mt. Erebus DOVEs are now visited more frequently yet others still remain pristine environments and there is a need to determine which caves are best suited for future microbiological research through a microbial community survey to determine if there has been anthropogenic intrusion on these communities. Since the earliest days of Mt. Erebus cave research, ice caves were thought of as naturally protected environments to be used for the placement of experiments, storing gear and even food. There is even the possibly that members of Antarctic Heroic Age explorers from either the Nimrod Expedition (1907–1909) with the first ascent in 1908 or the Terra Nova Expedition (1910–1913) with the highest camp up to that time, left food or materials near the caves. Establishment of McMurdo Station in 1956 by the US Navy helped increase the access to Mt. Erebus and continuous research began in the early 1970’s. One prominent Mt. Erebus cave, Warren Cave, is located in a logistically particularly strategic location, on a straight line between a recently discovered location of a camp by the Terra Nova Expedition (December 1912) and the summit of Mt. Erebus. This site is very close to Lower Erebus Hut, the operational base for the bulk of current research on Mt. Erebus.

The role of fungi in the DOVE communities is a new field of research and this is the first report of a fungal community associated with an Antarctic fumarole DOVE habitat.

## 2. Experimental Section

**Figure 1:**
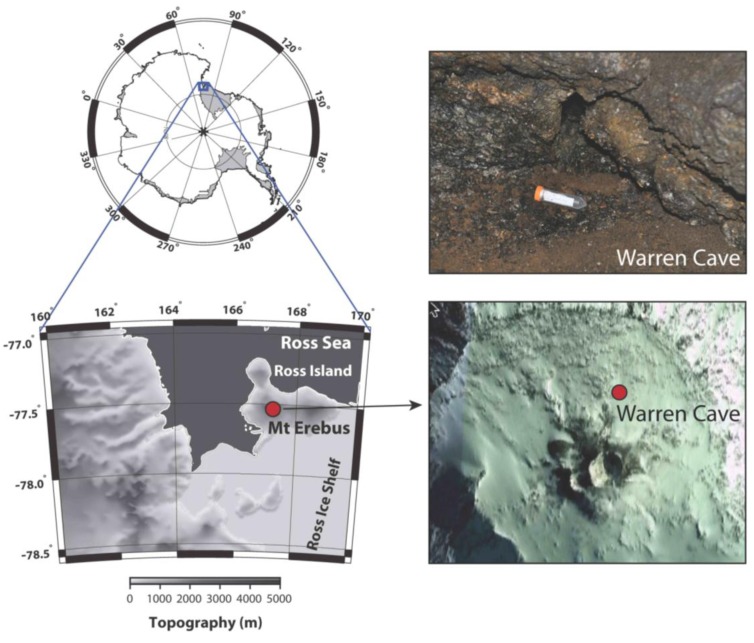
Warren Cave near Mt. Erebus Summit. The map shows the upper section of Mt. Erebus and the location of Warren Cave. The insert photo shows the site where the sample was taken.

Sample site: Warren Cave on Mt. Erebus, Antarctica (77° 31.003 S; 167° 09.884 E) (Figure 1) has been visited by researchers annually over the past decade for the study of volcanic CO_2_ emissions and temperature fluctuations by the Mount Erebus Volcano Observatory (MEVO) [4]. Warren Cave maintains a remarkably constant temperature. Temperatures in the fumarole studied were 18.5 °C inside the soil and 14.5 °C above the soil.

Sample collection: Soil substrate collected in 2010 from Warren Cave was taken from within a fumarolic vent issuing warm gas from beneath a protruding rock consisting of a patch of soil made up largely from coarse sand and fine gravel sized rock fragments (location labeled “GV 1” in [4]). The sample (Identification number: 10G439-WC) consisted of several pooled 10–15 g scoops collected aseptically in a sterile 50 mL tube. The sample was transported to the US at −20 °C. Soil temperature at the time of collection was 18.5 °C, the air temperature was 14.6 °C and soil pH was 5.2.

Soil analysis: Both soil moisture and carbon analyses were conducted by the University of Maine Analytical and Soil Testing Laboratory (Orono, ME, USA). Soil moisture was determined by gravimetric method at the time of soil drying for carbon analysis. Both total carbon and organic carbon were determined using the dry combustion method [5] with an Leco CN-2000 Carbon/Nitrogen analyzer.

Nucleic acid extraction and analysis: DNA from 1.62 g of the soil pellet was extracted using a ZR Soil Microbe Midi kit (Zymo Research, Irvine, CA, USA). The soil pellet was extracted 2× and the DNA was pooled. Three pellets were extracted for each clone library. Specific ITS region amplicons were produced by PCR (100 ng/reaction) in 25 μL reactions using Illustra PuReTaq Ready-To-Go™ PCR Beads (GE Lifesciences, Piscataway, NJ, USA). PCR primer set ITS5/ITS4 [6] was used to target the ITS region for clone library construction. Initial denaturation was for 2 min at 95 °C and 35 cycles with a PTC-200 thermal cycler (MJ Research, Watertown, MA, USA) under the following conditions: 30 s at 95 °C, 30 s at 52.3 °C, 1 min at 72 °C with a final 72 °C 10 min extension. The resulting PCR products were cleaned prior to cloning using Promega SV Gel and PCR Clean-up System (Promega, Madison, WI, USA). Three PCR reactions were produced from each soil pellet DNA aliquot and pooled prior to library construction. Two ITS clone libraries ligations were produced. PCR products from multiple (4–8) clean PCR reactions (*i.e.*, showing only bands within expected ITS size ranges) were pooled, and purified using a PCR purification kit (QIAGEN, Valencia, CA, USA). Libraries were generated using a TOPO TA cloning kit and chemically competent *Escherichia coli* TOP10F cells (Invitrogen, Carlsbad, CA, USA). The High Throughput Genomic Unit (HTGU—University of Washington, Seattle, WA, USA) preformed the transformations, clone selection and sequencing using the vector T7 primer. The resulting data were screened for (1) poor quality sequence (below 80% quality) and (2) short sequences (shorter than 400 bp) using Sequencher v 5.0.1 (Gene Codes Corp.) and these sequences were eliminated from further analysis. Sequences that passed the first two steps were then screened for chimeric sequences using Chimera Checker [7] and chimeric sequences were eliminated from further analysis. Each remaining sequence was reviewed by hand and compared with NCIB database (BLAST and Tree Builder) for taxonomic assignments. Based on suggestion of Fell and coworkers [8] sequence homology of >98% were considered to be the same species. GenBank accession numbers are shown in Table 1 and can be accessed through GenBank. The closest match isolates selected for Table 1 are comprised only of fungi that have been cultured. 

**Table 1:** Clones identified from Warren Cave with GenBank accession numbers and closest matches. Asterisks identify clones that fall below the 98% species identification threshold.

Warren Cave Clone Species	GenBank Accession Number	GenBank Closest Match	% match	GenBank Closest Match Species
*Acremonium implicatum*	KC785536	JQ692168	99%	*Acremonium implicatum*
* *Acremonium* sp.	KC785537	AB540571	94%	*Acremonium cereale*
*Alternaria alternata*	KC785538	AF218791	99%	*Alternaria alternata*
*Aspergillus penicillioides*	KC785539	HQ914939	99%	*Aspergillus penicillioides*
*Aureobasidium pullulans*	KC785542	FN868454	99%	*Aureobasidium pullulans*
*Aureobasidium* sp.	KC785543	HQ631013	99%	*Aureobasidium* sp. TMS-2011
*Candida zeylanoides*	KC785544	EF687774	100%	*Candida zeylanoides*
*Cladosporium* sp. 1	KC785545	HQ631003	100%	*Cladosporium* sp. TMS-2011 voucher
*Cladosporium grevilleae*	KC785546	JF770450	99%	*Cladosporium grevilleae*
*Cladosporium* *sphaerospermum*	KC785547	JQ776537	98%	*Cladosporium* *sphaerospermum*
*Clavispora lusitaniae*	KC785548	EU149777	99%	*Clavispora lusitaniae*
*Cochliobolus lunatus*	KC785549	HQ607915	100%	*Cochliobolus lunatus*
*Cyphellophora laciniata*	KC785550	EU035416	99%	*Cyphellophora laciniata*
*Epicoccum nigrum*	KC785551	HQ607859	100%	*Epicoccum nigrum*
*Erysiphe polygoni*	KC785552	AF011308	99%	*Erysiphe polygoni*
*Gibellulopsis nigrescens*	KC785553	KC156644	99%	*Gibellulopsis nigrescens*
*Hansfordia sp.	KC785554	HQ914948	96%	*Hansfordia* sp.
*Lewia infectoria*	KC785556	AY154718	99%	*Lewia infectoria*
*Myrothecium verrucaria*	KC785557	FJ235085	99%	*Myrothecium verrucaria*
*Penicillium oxalicum*	KC785558	JX231003	99%	*Penicillium oxalicum*
*Pezizomycotina sp.	KC785559	EU167561	96%	*Pleiochaeta ghindensis*
*Phaeococcomyces nigricans*	KC785560	AY843154	99%	*Phaeococcomyces nigricans*
*Phaeosphaeria* sp.	KC785561	HQ631018	99%	*Phaeosphaeria* sp. 1 TMS-2011 voucher
*Phialosimplex sp.	KC785562	GQ169326	93%	*Phialosimplex chlamydosporus*
*Pleosporales* sp.	KC785563	HQ207041	100%	*Pleosporales* sp. 24 PH
*Saccharomyces cerevisiae*	KC785564	AY939814	99%	*Saccharomyces cerevisiae*
*Tetracladium* sp. 1	KC785565	JF911760	98%	*Tetracladium* sp. QH32
*Tetracladium* sp. 2	KC785555	AB776690	99%	*Tetracladium* sp. SMU-1
*Toxicocladosporium irritans*	KC785566	EU040243	99%	*Toxicocladosporium irritans*
*Verticillium dahliae*	KC785567	HQ839784	99%	*Verticillium dahliae*
*Volutella colletotrichoides*	KC785568	AJ301962	100%	*Volutella colletotrichoides*
*Ceriporiopsis subvermispora*	KC785569	FJ713106	99%	*Ceriporiopsis subvermispora*
*Cryptococcus wieringae*	KC785570	FN824493	99%	*Cryptococcus wieringae*
*Cystofilobasidium macerans*	KC785572	AF444317	100%	*Cystofilobasidium macerans*
*Endophyte* sp.	KC785573	EU977202	99%	*Fungal Endophyte* sp. P807B
*Exidia glandulosa*	KC785574	AY509555	99%	*Exidia glandulosa*
*Exobasidium sp.	KC785575	EU784219	92%	*Exobasidium rhododendri*
*Filobasidium floriforme*	KC785576	AF190007	99%	*Filobasidium floriforme*
*Ganoderma applanatum*	KC785577	JX501311	99%	*Ganoderma applanatum*
*Glaciozyma watsonii*	KC785578	AY040660	99%	*Glaciozyma watsonii*
*Hymenochaete sp.	KC785579	JN230420	97%	*Hymenochaete corrugata*
*Hyphodontia rimosissima*	KC785580	DQ873627	99%	*Hyphodontia rimosissima*
*Irpex lacteus*	KC785581	EU273517	99%	*Irpex lacteus*
*Malassezia globosa*	KC785582	KC152884	99%	*Malassezia globosa*
*Malassezia restricta*	KC785583	EU400587	99%	*Malassezia restricta*
*Malassezia sp.	KC785585	KC141977	82%	*Malassezia sympodialis*
*Mycena* sp.	KC785587	JQ272379	99%	*Mycena* sp. 1 RB-2011
*Peniophora lycii*	KC785588	JX046435	99%	*Peniophora lycii*
*Phanerochaete sp.	KC785589	GU934592	96%	*Phanerochaete* sp. 853
*Polyporus sp.	KC785590	AF516599	97%	*Polyporus tuberaster*
*Resinicium bicolor*	KC785591	DQ826534	99%	*Resinicium bicolor*
*Rhodotorula mucilaginosa*	KC785592	HQ702343	99%	*Rhodotorula mucilaginosa*
*Sistotrema brinkmanii*	KC785594	DQ899095	99%	*Sistotrema brinkmannii*
*Skeletocutis chrysella*	KC785595	FN907916	99%	*Skeletocutis chrysella*
*Sporobolomyces* sp.	KC785596	EU002899	99%	*Sporobolomyces* sp.
*Stereum sanguinolentum*	KC785597	AY089730	99%	*Stereum sanguinolentum*
*Stereum sp.	KC785598	FN539049	91%	*Stereum rugosum*
*Trametes cubensis*	KC785599	JN164923	99%	*Trametes cubensis*
*Trichaptum sp.	KC785600	U63473	95%	*Trichaptum biforme*
*Ustilago sp.	KC785601	AY740170	97%	*Ustilago drakensbergiana*
*Ustilago tritici*	KC785602	JN114419	99%	*Ustilago tritici*

Community analysis: The resulting passed sequences were classified into groups based on their phyla. Each group of sequences was aligned using MUSCLE web server alignment [9]. The alignments were used to create phylogenetic trees through the *Seaview* software program, version 4.3.1. A rooted neighbor-joining distance tree was generated, for each phyla separately (Ascomycetes and Basidiomycetes), based on nucleotide positions of the ITS region of the 5.8S gene. Bootstrap values were based on 100 replicates. GenBank accession numbers were listed for the outgroup sequences. 

## 3. Results and Discussion

**Figure 2:**
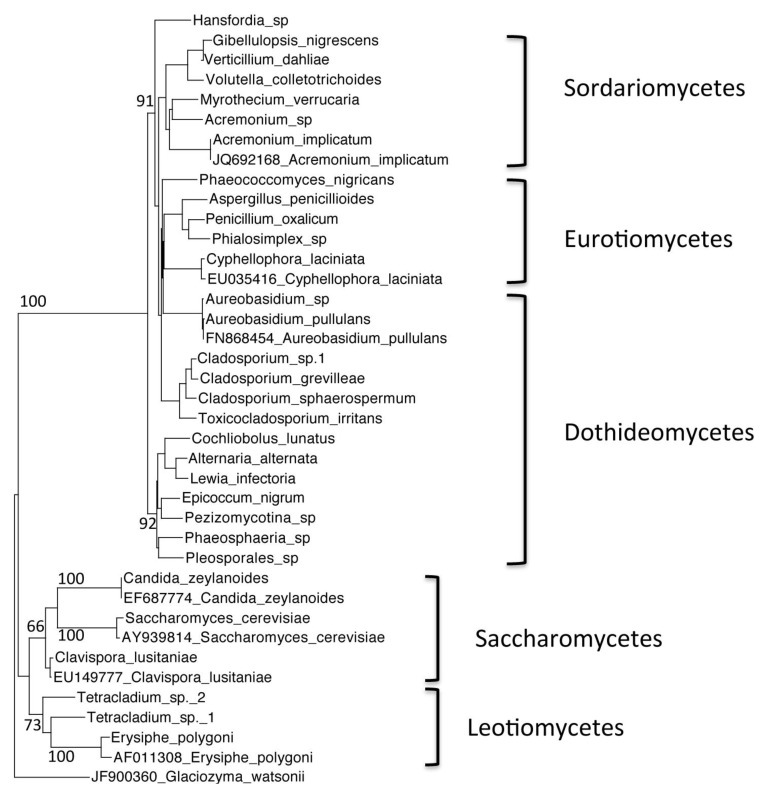
Phylogenetic tree of Warren Cave species belonging to the phylum Ascomycota, obtained by neighbor-joining analysis of the Internal Transcribed Spacer (ITS) region of the 5.8S rDNA gene, with 100 full heuristic replications. Bootstrap values are as indicated on the tree. *Glaciozyma watsonii* sequence obtained from GenBank was used as the outgroup, with the GenBank accession number listed. *Candida zeylanoides*, *Saccharomyces cerevisiae. Clavispora lusitaniae*, *Cyphellophora laciniata*, *Acremonium implicatum*, *Aureobasidium pullulans* and *Erysiphe polygoni* sequences obtained from GenBank were used as closest relative reference sequences, with GenBank accession numbers listed.

**Figure 3:**
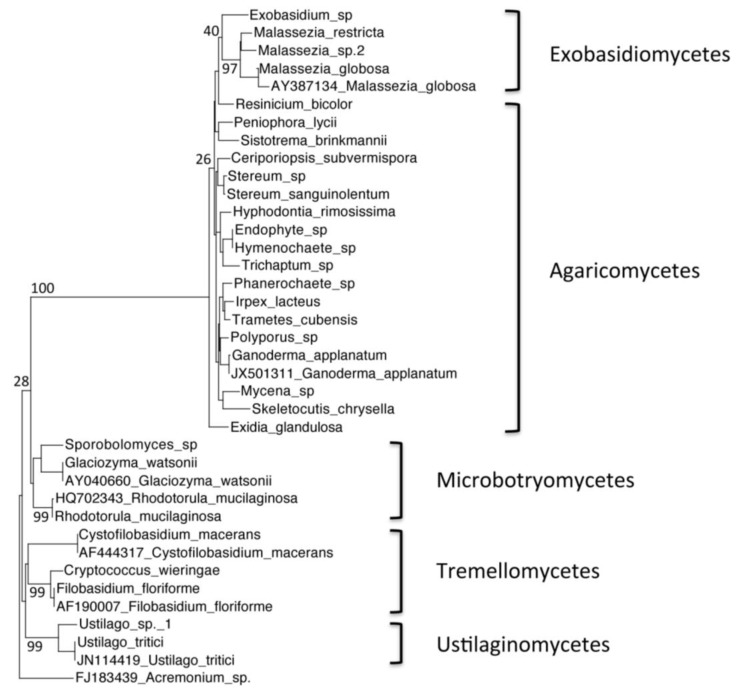
Phylogenetic tree of Warren Cave species belonging to the phylum Basidiomycota, obtained by neighbor-joining analysis of the Internal Transcribed Spacer (ITS) region of the 5.8S rDNA gene, with 100 full heuristic replications. Bootstrap values are as indicated on the tree. *Acremonium sp.* sequence obtained from GenBank was used as the outgroup, with the GenBank accession numbers listed. *Ustilago tritici*, *Glaciozyma watsonii*, *Rhodotorula mucilaginosa*, *Ganoderma applanatum*, *Malassezia globosa*, *Cystofilobasidium macerans* and *Filobasidium floriforme* sequences obtained from GenBank were used as closest relative reference sequences, with GenBank accession numbers listed.

We investigated the fungal diversity in Warren Cave though clone libraries. Soil substrate extraction was used to concentrate the fungal portion of the community prior to total DNA extraction. The habitat was highly oligotrophic with only 126 μg/g organic carbon (151 μg/g total carbon) and relatively moist with 50% soil moisture. Overall fungal diversity was moderate [10] with 266 fungal ITS clone sequences representing a total of 61 species. All were within the Ascomycota (Figure 2) and Basidiomycota (Figure 3) phyla, with no Chytridiomycota or Zygomycota represented. Near equal distribution of Ascomycota and Basidomycota taxa were represented in the Warren Cave clone libraries (31 Ascomycota/30 Basidomycota) unlike in nearby McMurdo Dry Valley habitats where Basidomycota dominate, especially in arid habitats [11,12,13]. The data in Table 1 show that a vast majority of clones found in Warren Cave can be identified to the species level (80%). Most of the remainder are relatively close matches with only one, a potential *Malassezia* species, with the closest match below 90% (82% identity with *Malassezia sympodialis*). The clones shown in this work are those that passed the criteria listed in the methods, yet within some of the chimeric clones (and not included in this analysis) DNA fragments of other organisms were found. These fragments were from organisms associated with humans. The most abundant of these were most closely identified as cabbage, soybeans, cereal grains and buckwheat. Interestingly, one of the dominant organisms found in the McMurdo Dry Valley soils, nematodes [14] were absent from these clone libraries, even in fragments.

Data on fungal communities in Antarctica still remain quite incomplete, however, it is possible to draw comparisons between our data from a Mt. Erebus DOVE to one other extreme environment in the McMurdo area, the nearby McMurdo Dry Valleys soils. The McMurdo Dry Valley soils are also highly oligotrophic, with some of the lowest organic carbon levels reported [15] and similar to those reported here. Their soil communities experience low temperature and rapid temperate swings, high UV radiation and desiccation [16], while the Mt. Erebus DOVEs have relatively moderate and constant temperature, no light, thus no UV radiation and a moist environment. In addition, many of the soils in the McMurdo Dry Valleys are basic with pH ranging up to pH10 [11], while this Warren Cave site was slightly acidic (pH 5.2). Therefore it is not surprising that the fungal communities are different. The typical number of fungal species found in any one soil community isolated from the McMurdo Dry Valleys is low, often below ten [11,12] whereas the fungal diversity found in Warren Cave was found to be much higher (61 species). The relative higher number of Ascomycota taxa found in Warren Cave compared with other studies in the McMurdo Dry Valleys may reflect the more stressful condition found in the latter. Yeast species in studies of the McMurdo Dry Valleys were dominated by basidiomycetous species (89%), most particularly those from the genus *Cryptococcus* (33%) [12]. The dominance of *Cryptococcus* species in soil, particularly arid soil, has been ascribed to their ability to produce polysaccharide capsules [17]. In contrast, only two *Cryptococcus* species were found in Warren Cave. Further, *Glaciozyma watsonii* has been isolated numerous times from soil from Continental Antarctic soil [12,18,19] and can be an abundant member of the McMurdo Dry Valley soil community, but are represented by only six clones (4.9%) in Warren Cave. *Rhodotorula mucilaginosa* has also been cultured from some of the most dry and cold locations in Antarctica, such as Sponsors Peak (03SP24) and a peak above Niebelungen Valley, in the Asgard Range (03NB35) [12] yet was represented by only 2 clones (1.6%) of the Warren Cave libraries.

**Figure 4:**
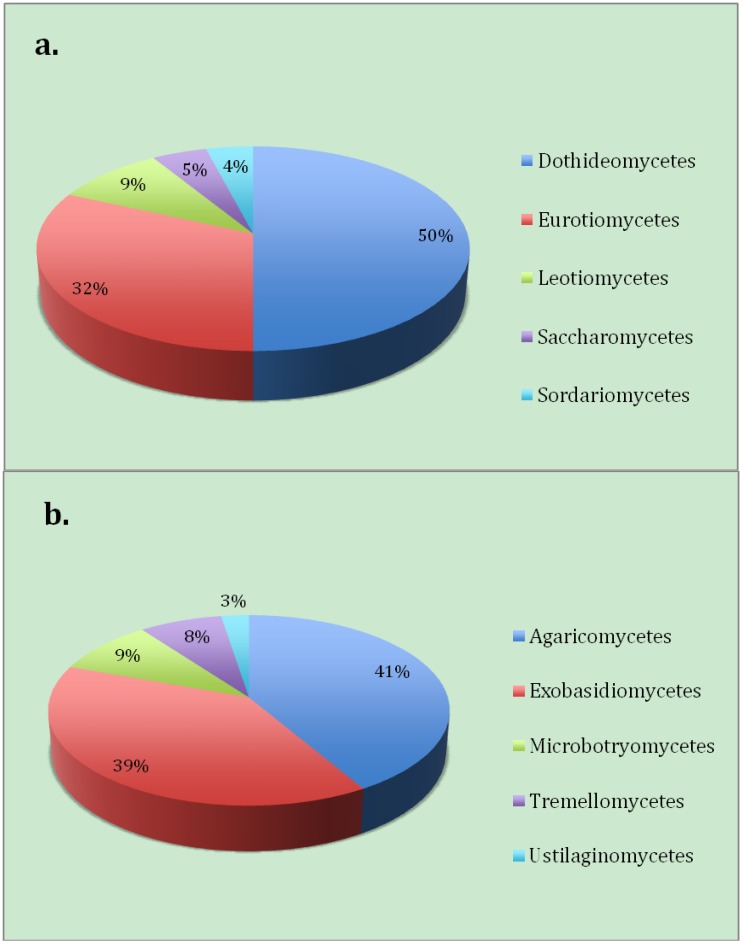
The proportion of the number of fungal clones in this study by class belonging to (**a**) Ascomycota and (**b**) Basidiomycota.

*Aureobasidium pullulans* a yeast-like fungus, *Aspergillus penicillioides* and *Alternaria alternata* were found to be the dominating members of the Ascomycota (Figure 4a) from the Warren Cave fungal community. All of these species are cosmopolitan and have been isolated numerous times from the Antarctic [20]. The most dominant Basidiomycota taxa found (Figure 4b) were *Malassezia* sp. (37% of the clones), yeasts most typically found associated with animals. Although *Malassezia* has also been identified from a clone library originating from McMurdo Dry Valleys desert soils of Taylor Valley, Antarctica (near a highly used pathway) [13] two of the species found in this study (*M. globosa* and *M. restricta*) are know to require lipids for growth and are common in human dandruff and seborrheic dermatitis [21]. This high proportion of human associated yeasts represented in the Warren Cave clone libraries suggests human contamination of the site. The second most abundant taxa of the Basidomycota was a *Peniophora* species, a member of a genus most known for wood rot [22].

Although several fungal species found in Warren Cave are cosmopolitan, some have been shown to be capable of efficient colonization on minerals and sterile soil [23], abilities that enhance the probability that these organisms are active members of the DOVE community. For example *Irpex lactecus*, a white rot fungus, has been suggested as a potential bioremediation agent precisely because of its ability to utilize minerals and thrive in sterile soil. [24]. *Aureobasidium pullulans* has been isolated often from Antarctica, including McMurdo Dry Valley soil and has been found on the inner part of the Chernobyl inner containment system [25]. Both of these species may be early colonizers of oligotrophic habitats and potentially native Antarctic DOVE inhabitants.

## 4. Conclusions

The Warren Cave fungal community appears to have been influenced by humans. The cave has been visited throughout the years by both researchers and casual visitors. Evidence of human involvement in Warren Cave is supported by the findings of several *Malassezia* species. Finding species in a clone library alone does not necessary mean that they are active members of the community and more research will have to be carried out to determine their role, if any, in the Warren Cave soil community. As a result, it may not be surprising that Warren Cave is somewhat biologically compromised, and currently contains items that may impact and alter cave microbial communities (e.g., metal or bamboo stakes, instruments such as temperature recorders, batteries metal wires, *etc*.). It is clear that some of these practices profoundly impact the local microbial communities.

Even though Warren Cave has had human impact on its microbial community, other caves around the summit of Mt. Erebus remain untouched and therefore are potentially very valuable natural research laboratories for the study of DOVEs. The U.S. National Science Foundation has begun a procedure to develop a code of conduct for entering ice caves on Mt. Erebus. One of the first steps in this effort should be to determine which caves remain pristine and therefore are suitable candidates for microbial DOVE research.

Our data show that microbial communities in Mt. Erebus Ice Cave DOVEs contain diverse and specialized fungal communities that are likely to form a complex microbial foodweb that is independent from photosynthesis and primarily uses energy from chemolithotrophic metabolic processes. Very little is known about their function in these ecosystems, but several species are known to grow on mineral substrates in sterile soil supporting an active role. In addition, our study shows that a survey of fungi is a very sensitive indicator identifying the potential of human disturbance of these environments.
